# Optimal Screening and Detection Strategies for Cervical Lesions: A Retrospective Study

**DOI:** 10.7150/jca.96128

**Published:** 2024-05-13

**Authors:** Yueming Yang, Lijiang Xu, Songhua Yuan, Jin Lv, Pengchen Chen, Wei Wang

**Affiliations:** 1 Department of Obstetrics and Gynecology, The First People's Hospital of Foshan, Foshan, 528000, Guangdong, China.; 2 Department of Pathology, The First People's Hospital of Foshan, Foshan, 528000, Guangdong, China.; 3 Guizhou Provincial Key Laboratory of Pathogenesis and Drug Research on Common Chronic Diseases, Guizhou Medical University, Guiyang, 550000, China.; 4 Department of Pathophysiology, Guizhou Medical University, Guiyang, 550000, China.

**Keywords:** cervical lesion, early-screening, disease diagnosis, TruScreen

## Abstract

**Background:** Cervical cancer is the fourth most common cancer among women worldwide. Cervical cancer usually develops from human papillomavirus (HPV) infection, which leads to cervical intraepithelial neoplasia (CIN1/2/3) and eventually invasive cervical cancer. Therefore, early-screening and detection of cervical lesions are crucial for preventing and treating cervical cancer. However, different regions have different levels of medical resources and availability of diagnostic methods. There is a need to compare the efficiency of different methods and combinations for detecting cervical lesions and provide recommendations for the optimal screening and detection strategies.

**Methods:** The current clinical methods for screening and detection of cervical lesions mainly include TruScreen (TS), Thinprep cytologic test (TCT), HPV testing, and colposcopy, but their sensitivity and specificity vary and there is no standard protocol recommended. In this study, we retrospectively reviewed 2286 female samples that underwent cervical biopsy and compared the efficiency of different methods and combinations for detecting cervical lesions.

**Results:** HPV screening showed the highest sensitivity for identifying women with CIN2+ cervical lesions compared with other single methods. Our results also showed the importance and necessary of the secondary diagnostic test like TCT and TS as a triage method before colposcopy examination and guided biopsy.

**Conclusions:** Our study provides recommendations for the optimal screening and detection strategies for cervical lesions in different regions with different levels of development. As a non-invasive, easily operated, and portable device, TS is a promising tool to replace TCT for detecting cervical lesions in the health care center with insufficient medical resources.

## Introduction

Cervical cancer is one of the most preventable and treatable types of cancer, yet it remains a leading cause of death among women around the world. According to the latest report from World Health Organization (WHO), cervical cancer accounted for an estimated 604,000 new cases and 342,000 deaths in 2020, and more than 90% of the death burden occurring in low- and middle-income countries 1. The main cause of cervical cancer is persistent infection with high-risk human papillomavirus (HR-HPV) types, which can induce cervical intraepithelial neoplasia (CIN), a precancerous condition that can progress to invasive cervical cancer if left untreated 2-4. Therefore, effective cervical cancer screening and diagnosis of cervical lesions are crucial for preventing cervical cancer and reducing mortality.

For screening and detecting of cervical lesions, several methods are available, such as visual inspection of acetic acid or lugol's iodine (VIA/VILI), pap smear, TruScreen (TS), Thinprep cytologic test (TCT), colposcopy, and so on 5-8. These methods have different advantages and limitations in terms of sensitivity, specificity, cost, and accessibility. For example, pap smear has been used as the standard diagnostic method for cervical cancer screening, but it was replaced by HPV screening because of its higher sensitivity 9. However, HPV screening has showed a low specificity and may result in overdiagnosis and overtreatment of the patients with transient HPV infections or low-grade cervical lesions 10. In the areas with insufficient medical resources, TCT diagnosis is not available due to the lack of pathology laboratory. Colposcopy examination and cervical biopsy under colposcopy are critical in the detection of cervical lesions, and the detection quality largely depends on the skill and experience of the colposcopy specialist.

Cervical cancer screening guidelines have been released by various countries and organizations in recent years. In 2021, WHO recommended that women aged 30 to 50 undergo HPV DNA testing every 5-10 years or cytology or VIA as alternative tests 11. The European Commission also advocates HPV DNA testing for women aged 30 to 65 every 5 years 12. In the United States, HPV testing is the preferred primary screening method for women aged 25 to 65. Where primary HPV testing is unavailable, the co-testing with HPV and cytology or cytology alone is considered acceptable 13. In China, the co-testing with HPV and cytology is the most recommended screening method is the co-testing of HPV screening and cytology when medical resources are ample. In the absence of sufficient resources, HPV screening alone is recommended as the primary method, with cytology as a secondary option 14.

In recent years, artificial intelligence integrated algorithms have been introduced to help colposcopy specialist in diagnosis and biopsy 15,16. Moreover, the performance of these methods may vary depending on the prevalence and distribution of HPV types and the stage and grade of cervical lesions. In clinical practice, the patients with cervical lesion CIN2 or worse including CIN2, CIN3, and cancer (CIN2+) are at high risk of developing cancer and need active surveillance or treatment 17,18. Consequently, there is a need for more context-specific guidelines for the optimal screening and detection strategies for cervical lesions.

In this study, we aimed to compare the efficiency of different methods and combinations of methods for detecting cervical lesions in a large cohort of women who underwent cervical biopsy. We retrospectively analyzed the data of 2286 female samples and evaluated the sensitivity and specificity of TS, TCT, HPV screening, and colposcopy, as well as their combinations, for diagnosing CIN2+ cervical lesions. Based on our findings, we proposed the recommendations for the optimal screening and detection strategies for cervical lesions in different settings.

## Materials and Methods

### Study design and sample collection

**Figure 1:**
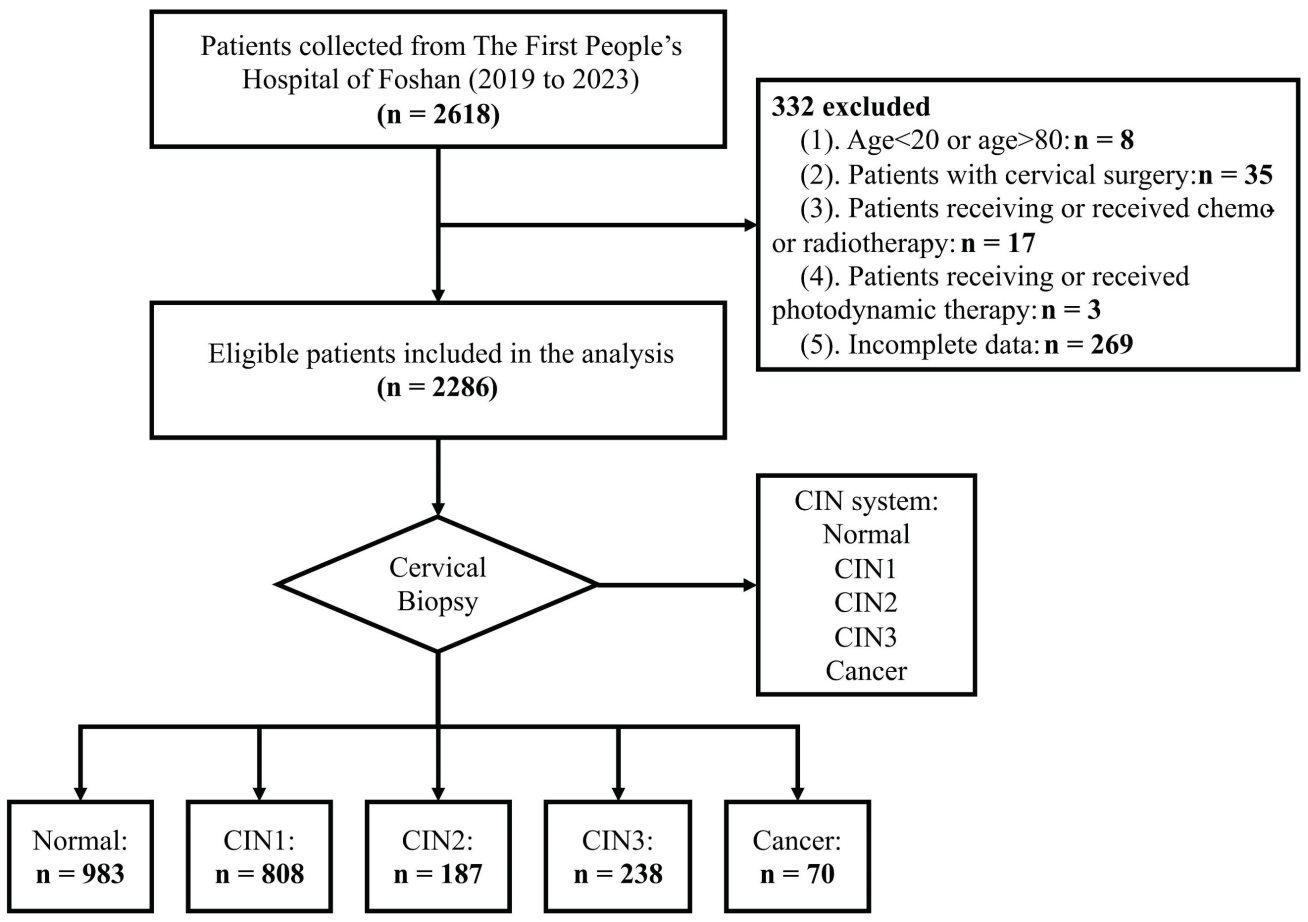
Flow chart showing the patient inclusion and exclusion criteria. CIN: cervical intraepithelial neoplasia.

We conducted a retrospective study that aimed to compare the efficiency of different methods and combinations for detecting cervical lesions among women who underwent cervical biopsy 19,20. We collected the clinical records of patients from The First People's Hospital of Foshan during the study period (January 2019 and December 2023). We included 2286 women aged 20 to 80 years who underwent cervical biopsy and had complete demographic and clinical data. We extracted the following data from the clinical records: TS results, cytological results, HPV screening results, colposcopy results, and cervical biopsy results. Women who had a history of cervical surgery, chemotherapy, radiotherapy, photodynamic therapy, or incomplete clinical records were excluded. The flowchart of the study population selection is shown in Figure 1.

### Laboratory Methods

TS is an electro-optical device that measures the electrical impedance of cervical tissue and provides a real-time result. We followed the operating procedures from the training manual for TS detection. The probe with the sensor was placed on the cervical surface in a fixed order of points, and 30 points were collected in total. For the type 3 transformation zone (TZ), at least four points were completed on the upper, lower, left and right sides of the cervical canal. TS detection reported binary diagnosis results, TS-negative and TS-positive.

TCT is a liquid-based cytology method that uses a thin layer of cells to detect abnormal morphology. The cytological examination was based on the Bethesda system (TBS) for diagnosis 21. The cytological results were divided into five categories, including negative for intraepithelial lesion or malignancy (NILM), atypical squamous cells of undermined significance (ASC-US), low-grade squamous intraepithelial lesion (LSIL), atypical squamous cells-cannot exclude high-grade squamous intraepithelial lesion (ASC-H), and high-grade squamous intraepithelial lesion (HSIL). The patients were placed in the lithotomy position, and the speculum exposed the entire vaginal cervix. After removing the excessive cervical secretions, a special cell collector was used to repeatedly smear and collect samples from the patient's cervix or vaginal wall. The samples were preserved in liquid-based cytology medium until analysis. Before sampling, it was confirmed that the patients had no vaginal medication or sexual intercourse for the previous 3 days.

HPV testing is a molecular method that detects the presence of HR-HPV types using polymerase chain reaction (PCR). We used a commercial kit (cobas HPV test, Roche Molecular Systems) to extract DNA from the samples and amplify the target regions of 14 HR-HPV types (namely 16, 18, 31, 33, 35, 39, 45, 51, 52, 56, 58, 59, 66 and 68). We detected the amplified products using a hybridization capture method and reported the positive ones as HPV infection.

Colposcopy is a visual examination of the cervix using a magnifying device and acetic acid. The colposcopy examination used the EDAN C6A colposcope. The main steps included: colposcopy examination, acetic acid white test, iodine test, and colposcopy-guided biopsy. The examination was performed by a colposcopy specialist following the 2011 IFCPC colposcopic terminology for the cervix 22. In accordance with the Lower Anogenital Squamous Terminology (LAST) system, the colposcopy results were classified into four categories, including normal, LSIL, HSIL, and cancer 23. The speculum fully exposed the cervix and the entire vagina. After removing the excess secretions, 5% acetic acid was applied to the cervix and vaginal wall. The observation was done under 6-20 times magnification for 2 minutes. Then, 3% compound iodine solution was applied to the entire cervix and vaginal wall. Multiple biopsies were performed on the acetic acid white epithelial area or the iodine test non-staining area. If no lesions were found, multiple random biopsies were performed and sent for pathological examination. The pathological results were considered as the gold standard.

The collected cervical tissue specimens were immediately fixed in 10% neutral buffered formalin for 16 to 24 hours, and then sent to the laboratory for embedding, sectioning, and diagnosis. The diagnosis was made by the senior histopathologists in a blind manner. We used the three-tiered CIN classification, and the results included: normal (including chronic cervicitis, cervical polyps, HPV infection-related lesions, etc.), CIN1 (lesions involving the lower 1/3 of the squamous epithelium), CIN2 (lesions involving the 2/3 of the squamous epithelium), CIN3 (lesions involving the whole layer of the squamous epithelium), and cancer (microinvasive carcinoma, squamous cell carcinoma, *in situ* adenocarcinoma, and adenocarcinoma) 24.

### Statistical Analysis

R software (http://www.R-project.org) was used for statistical analyses. We calculated the sensitivity (true positive rate, TPR) and specificity (true negative rate, TNR) of each method and the combined methods for detecting cervical lesions using 2x2 contingency tables (Table 1.):



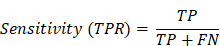





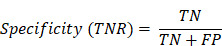



In above formulas, TP stands for true positive, TN stands for true negative, FN stands for false negative, FP stands for false positive. The performance of the single and combined methods was further assessed using the area under the area under the receiver operating characteristic (ROC) curve (AUC). We used the “*pROC*” package to plot the ROC curves and calculate the AUC values.

## Results

### Characteristics of Study Population

**Figure 2:**
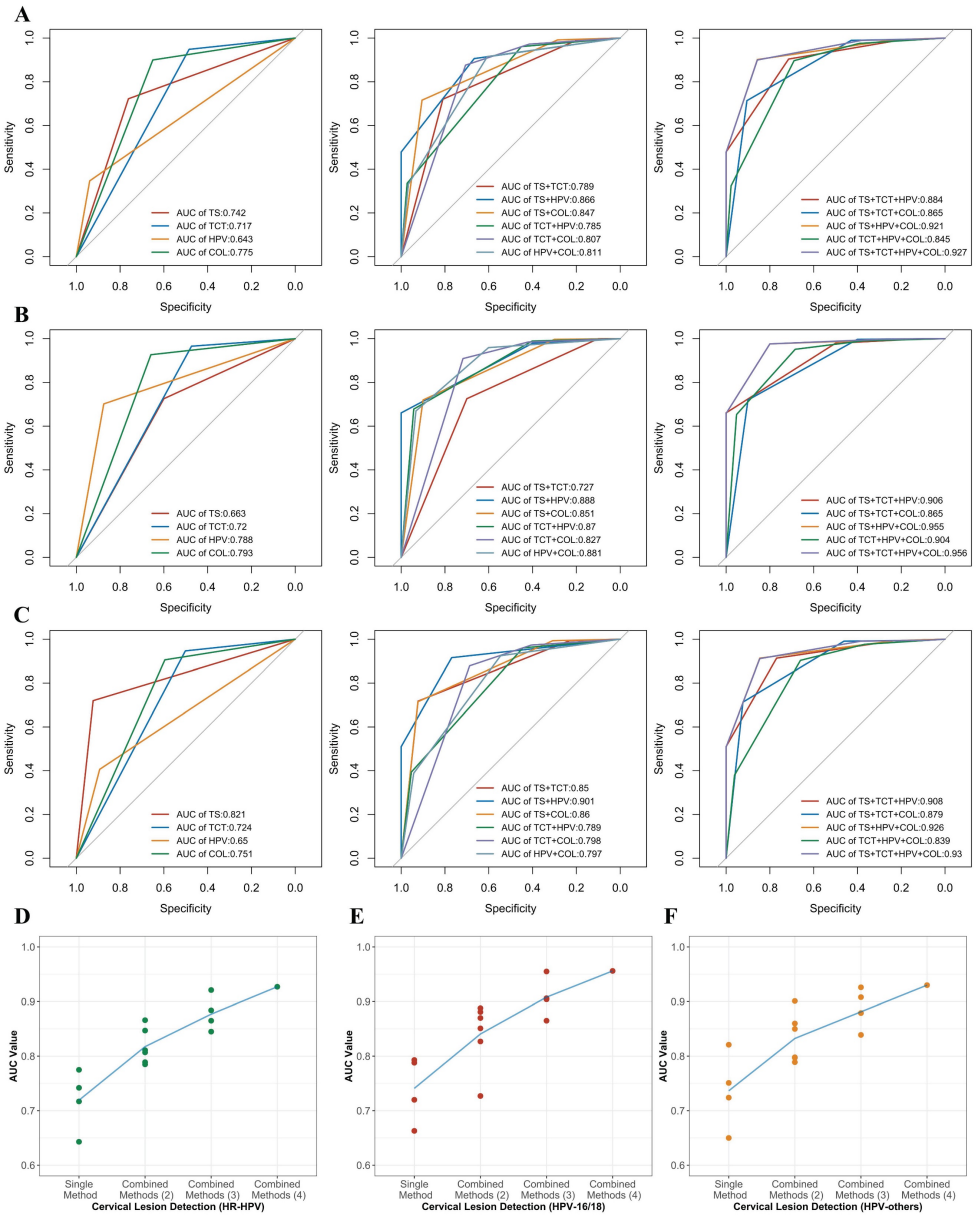
Representative diagnostic figures from multiple methods for detecting CIN2+ cervical lesions. NILM: intraepithelial lesion or malignancy; ASC-US: atypical squamous cells of undermined significance; LSIL: low-grade intraepithelial lesion; ASC-H: high-grade squamous intraepithelial lesion; HSIL: high-grade intraepithelial lesion; CIN: cervical intraepithelial neoplasia.

We compared the efficiency of different methods and combinations for detecting cervical lesions among 2286 women who underwent cervical biopsy at The First People's hospital of Foshan. The study population had a mean age of 41.3 ± 10.4 years, ranging from 20 to years. The majority of the population (66.0%, 1508 out of 2286) were aged between 30 and 45 years. A total of 489 patients were received TS detection, and 343 of them (70.1%) showed negative results. Cytologic records were available for 2200 patient, and NILM diagnoses were given to 947 patients (43.0%). The other cytological categories were as follows: ASC-US (21.8%, 479 out of 2200), LSIL (21.0%, 463 out of 2200), ASC-H (3.1%, 69 out of 2200), HSIL (10.7%, 236 out of 2200), and Cancer (0.3%, 6 out of 2200). The majority of patients (96.3%, 2202 out of 2286) were received HPV screening, and 1569 of them (71.2%) were positive for HR-HPV types. HPV types 16 and 18 accounted for 20.5% (452 out of 2202) of the study population. Lastly, 2279 patients underwent colposcopy examination and colposcopy-guided cervical biopsy, and 63 of them (2.8%) were diagnosed with cancer. The other colposcopy categories were as follows: Normal (38.7%, 881 out of 2279), LSIL (39.4%, 897 out of 2279), HSIL (19.2%, 438 out of 2279), and Cancer (2.8%, 63 out of 2279). The distribution of age, TS results, cytologic records, HPV status, colposcopy examinations, and cervical lesions are displayed in Table 2. The representative diagnostic figures from different detection methods were shown in Figure 2.

### Performance of different methods and combinations

**Figure 3:**
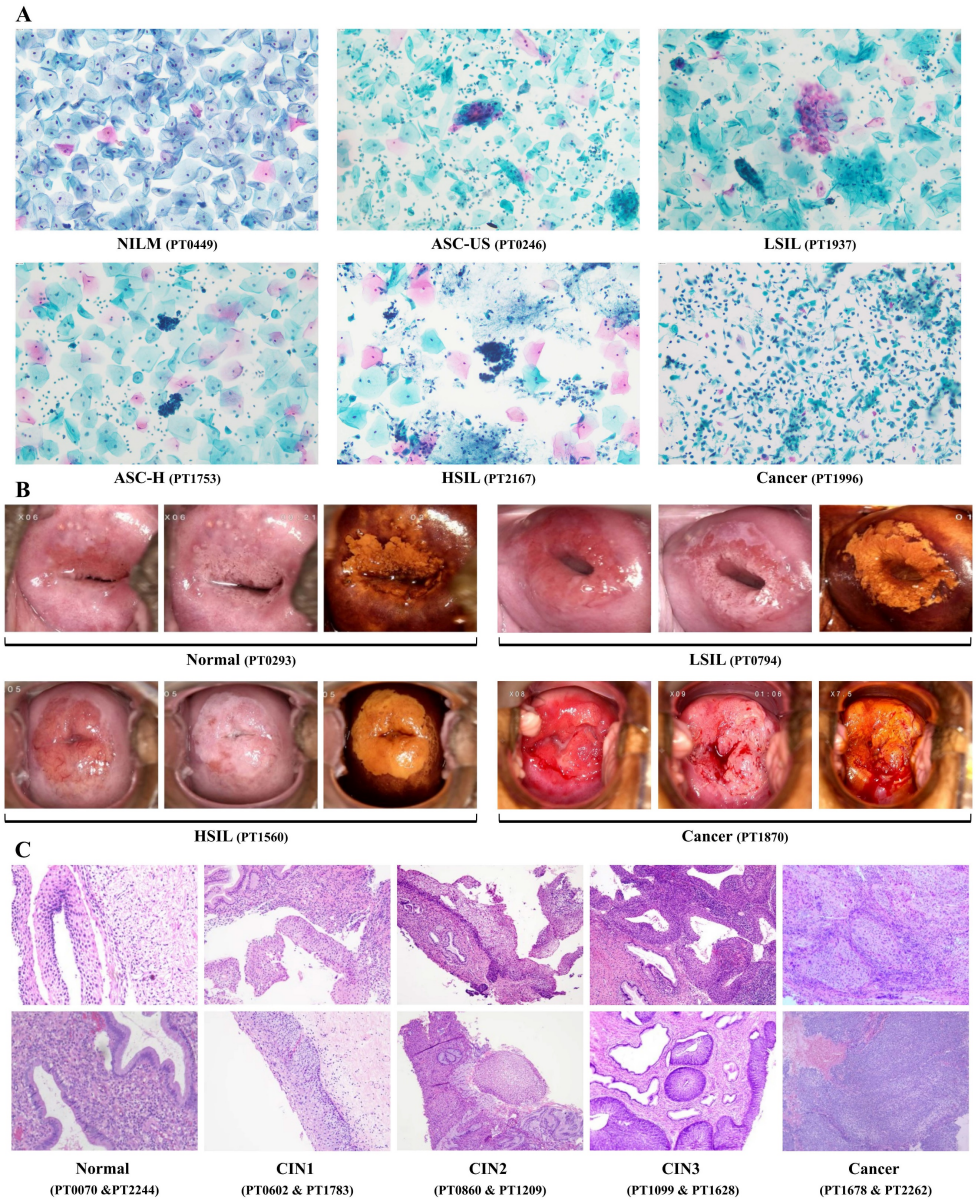
ROC analysis evaluating the performance of different methods and combinations for detecting CIN2+ cervical lesions with different HPV status: HR-HPV (A), HPV-16/18 (B), and HPV-others (C). The improved performances have been observed when the increase of the number of methods combined for detecting CIN2+ cervical lesions with different HPV status: HR-HPV (D), HPV-16/18 (E), and HPV-others (F). The average AUC values have been highlighted and linked by the blue line. ROC: receiver operating characteristic; AUC: area under the curve.

In the detection of cervical lesions, different methods were applied and their sensitivities and specificities are varied. In this section, we compared the sensitivities and specificities of different methods and combinations for detecting cervical lesions with different HPV status (Table 3 and Figure 3). We also performed ROC analyses to assess the discrimination performance of different methods and combinations. We found that the performance improved with the increase of the number of methods combined (Figure 3D, E and F). Among all the results, the combination of all four methods showed the highest AUC values for the women with different HPV status (HR-HPV, HPV-16/18, and HPV-others, respectively) in the detection of CIN2+ cervical lesions. The AUC values were 0.927 (95% CI: 0.881-0.972), 0.956 (95% CI: 0.910-1.000), and 0.930 (95% CI: 0.875-0.986), respectively.

However, it is unrealistic to conduct all examinations for detecting CIN2+ cervical lesions in either early-screening or diagnosis, especially in the economically undeveloped or developing areas where pathology laboratory is unavailable. Therefore, it is significant to select an optimal workflow for the detection of CIN2+ cervical lesions among multiple diagnostic tests. There are two different ways to handle the results from multiple diagnostic tests: serial testing and parallel testing^25^. Parallel testing (the “or” rule) reports a negative outcome only if all methods give negative predictions, which helps to improve the sensitivity of the testing scheme. Serial testing (the “and” rule) reports a positive outcome only if all methods give positive predictions, which helps to improve the specificity of the testing scheme.

The sensitivity and specificity of each method and combination for detecting cervical lesions are shown in Table 4. In the detection of cervical lesions CIN2+, HPV screening showed highest sensitivity of 93.9% for women with HR-HPV compared with other three single methods (Supplementary Table S1 and S2). For the combination of two methods, TS combined with TCT (the “and” rule) showed 100% specificity for identifying <CIN2 cervical lesions in women with HPV-16/18. TS combined with HPV screening (the “or” rule) showed 100% sensitivity for identifying CIN2+ cervical lesions in women regardless of their HPV status. For the combination of more than two methods, the multiple diagnostic tests all showed high sensitivity and specificity (more than 90%) for detecting CIN2+ cervical lesion. These results suggest that different methods and combinations have different strengths and limitations for detecting cervical lesions, and the optimal workflow may depend on the availability of resources and the HPV status of the women.

### Recommended workflows for cervical lesion detection

We proposed two recommended workflows for CIN2+ cervical lesion detection based on the performance, accuracy, and availability of different methods and combinations. The first workflow is the most recommended workflow when all detection methods are available. The second workflow is the recommendation without the TCT for the economically undeveloped or developing areas where pathology laboratory is not available. The advantages and disadvantages of the preferred diagnostic method were summarized in Table 5.

**Figure 4:**
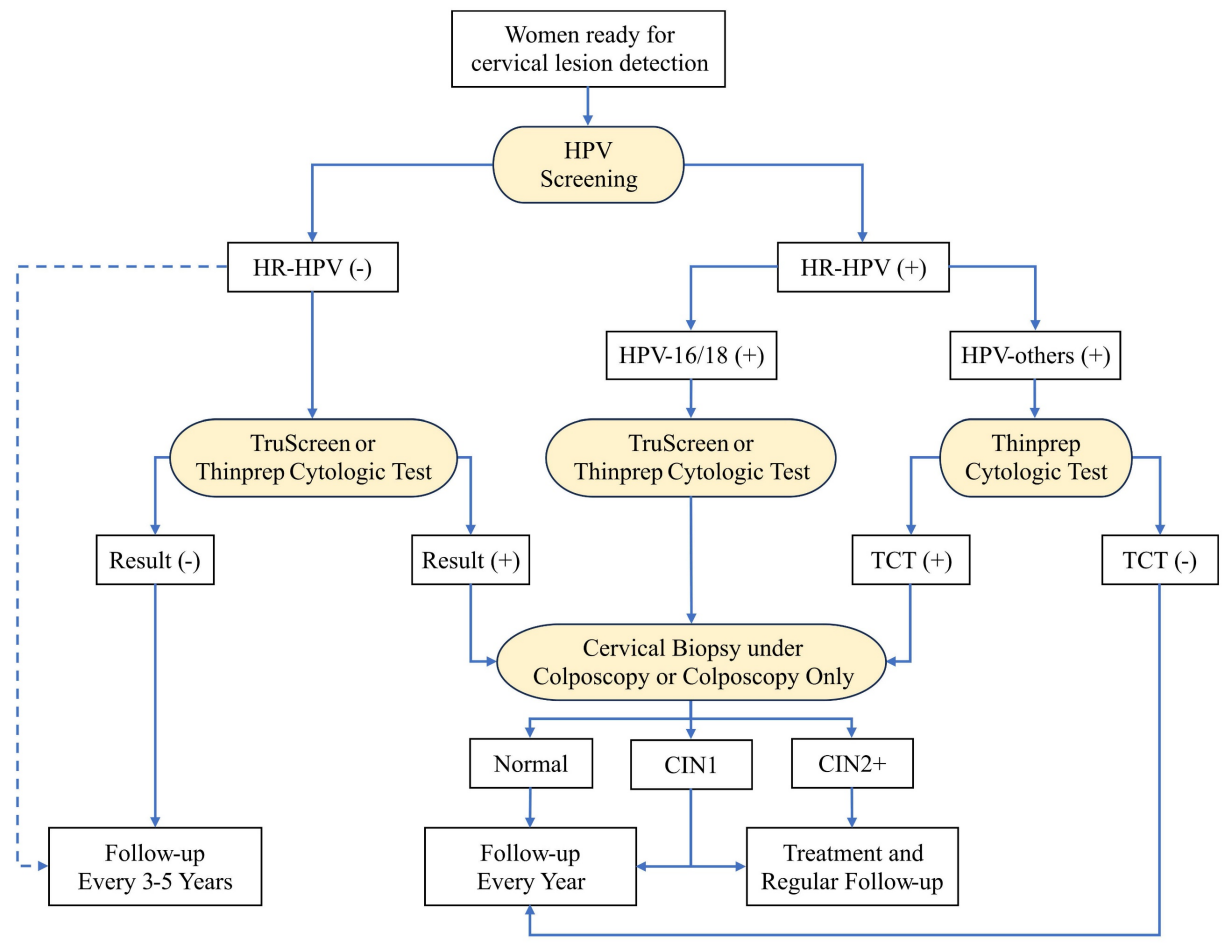
The optimal workflow for cervical lesion detection when all detection methods are available. The diagnostic methods are highlighted with yellow. The blue dashed line highlights the alternative direction for HR-HPV-negative patients. HPV: human papillomavirus; TCT: thinprep cytologic test; CIN: cervical intraepithelial neoplasia.

The first workflow is shown in Figure 4, which consists of four detection methods including HPV screening, TS, TCT, and cervical biopsy under colposcopy or colposcopy only. We recommended HPV screening as the primary method for early-screening of cervical lesions because it has the highest sensitivity compared with other single methods (Supplementary Table S3). HPV screening combined with TS or TCT is optional to lower the false positive rate. For women with HPV-16/18, either TS or TCT is the secondary diagnostic method, which significantly improves the specificity for detecting CIN2 cervical lesion compared to HPV screening only (Supplementary Table S4 and S5). For women with HPV-others, TCT is the recommended secondary diagnostic method, which shows better performance than additional TS test (Supplementary Table S5). The colposcopy is the follow-up test and cervical biopsy under colposcopy is needed for women who have suspicious lesion areas on colposcopy. The pathological results are considered as the gold standard to confirm the presence and extent of cervical lesions or cancer. This workflow is the optimal combination of multiple diagnostic methods for detecting CIN2+ cervical lesions, as it has the highest sensitivity, specificity, among all combinations, according to our results.

**Figure 5:**
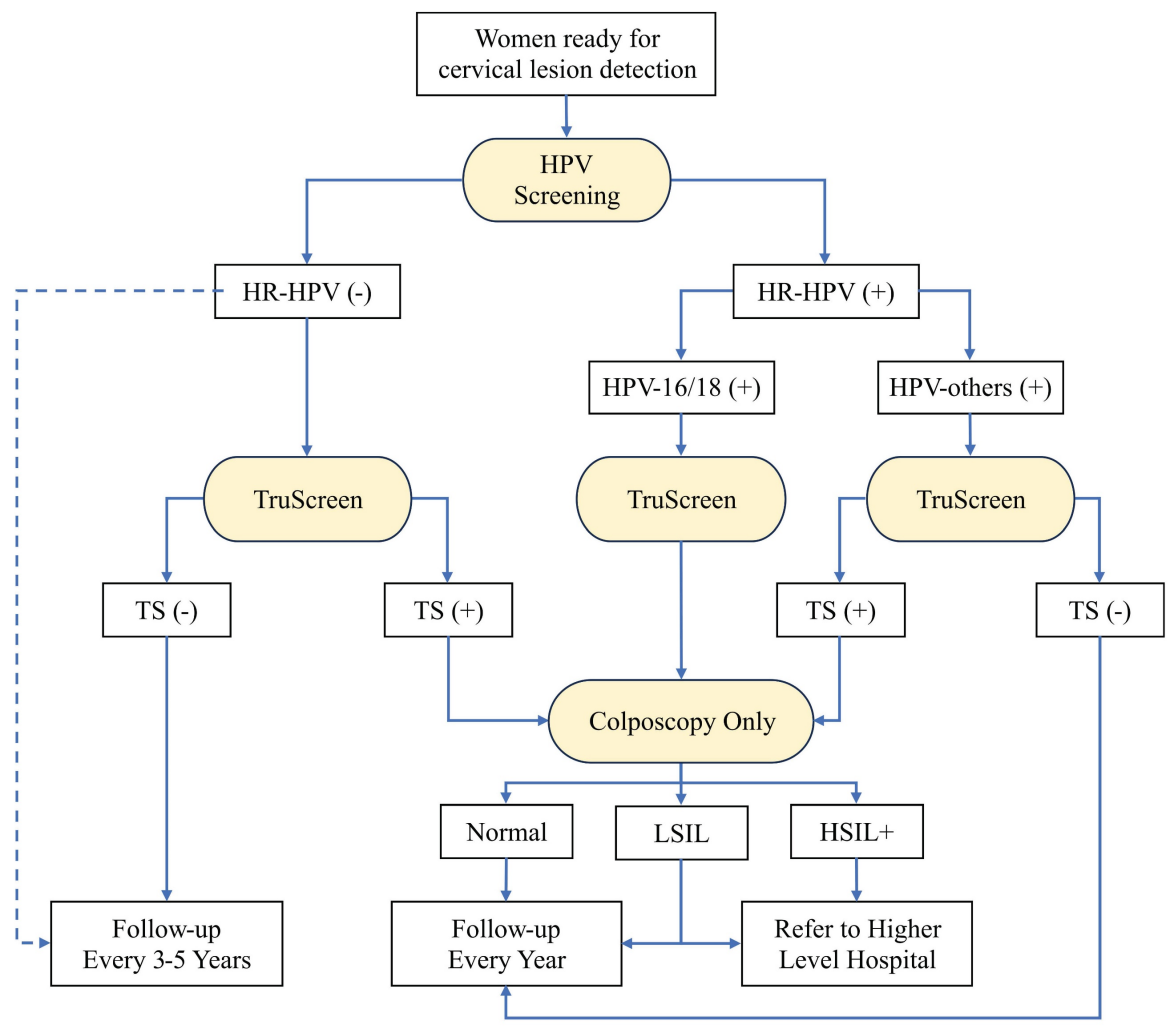
The recommended workflow for cervical lesion detection when pathology laboratory is not available. The diagnostic methods are highlighted with yellow. The blue dashed line highlights the alternative direction for HR-HPV-negative patients. HPV: human papillomavirus; TS: TruScreen; LSIL: low-grade squamous intraepithelial lesion; HSIL: high-grade squamous intraepithelial lesion.

The second workflow is shown in Figure 5, which consists of three detection methods including HPV screening, TS, and colposcopy. The HPV screening is recommended as the primary screening method for early-screening of cervical lesions like the first workflow (Supplementary Table S3). The HPV screening combined with additional TS test is still optional choice to achieve a higher true positive rate for early-screening of cervical lesions. TS is the secondary detection method for women with HPV-positive. This combination significantly increases the specificity from 40.7% to 91.6 for identifying <CIN2 cervical lesions compared with HPV screening only (Supplementary Table S4). As same as the first workflow, colposcopy is the follow-up diagnostic test for women with HPV-16/18 or TS-positive (HPV-others). Because of the lack of pathology laboratory, we recommend women diagnosed as LSIL or HSIL+ by colposcopy specialist to be referred to higher level hospital for further cervical biopsy. This workflow is the recommendation without TCT when pathology laboratory is not available, as it has a high sensitivity and specificity, according to our results.

## Discussion

In this retrospective study, we aimed to provide recommendations for the optimal screening and detection strategies for cervical lesions in regions with different levels of development. We compared the efficiency of different methods and combinations for detecting cervical lesions among 2286 women who underwent cervical biopsy at The First People's Hospital of Foshan. Our results indicated that HPV screening should be recommended as the primary method in early-screening because of its superior sensitivity in the identification of women with CIN2+ cervical lesions compared with other single methods. We recommend TS or TCT as the secondary diagnostic methods, depending on the HPV status of the patients and the availability of the pathology laboratory. The combination of HPV and additional diagnostic method significantly improves its specificity of disease diagnosis compared to HPV screening alone. Cervical biopsy under colposcopy or colposcopy only is the follow-up diagnostic test which is recommended for women with HPV-16/18 patients or TCT-positive or TS-positive diagnosis (HPV-others).

Other cytology-based methods are applied for cervical cancer screening, they are limited by the low sensitivity compared with HPV screening^9^,^26^. Therefore, HR-HPV screening has been used as the primary method for early-screening of cervical cancer in many countries 11-14,27,28. In our retrospective study, HPV screening showed the highest and second highest sensitivity (87.4% and 89.3%) for detecting CIN2+ cervical lesions for women with HPV-16/18 and HPV-others respectively (Supplementary Table S3). However, HPV screening also had a small portion of false negative findings (~10%), which could lead to missed diagnosis of cervical lesions. To address this problem, we recommend TS or TCT as an optional diagnostic step to further prevent missed diagnosis. The combination of HPV screening and additional method significantly improved the sensitivity compared to HPV screening alone. For tested patients, HPV screening plus TCT increased the sensitivity from 93.9% to 97.3% (p=0.0149), while HPV screening plus TS had no significant different in sensitivity (93.9% to 100.0%, p=0.6258) due to its limited statistical power. The additional diagnostic test of TS is still a recommended option because it had zero missed diagnosis.

Although HPV screening has been considered as the primary method in early-screening of cervical cancer, it cannot be ignored that its low specificity will lead to the overtreatment of the patients who showing false positive results 10. Therefore, the combination of multiple methods may improve the performance and accuracy of cervical cancer screening and detection. The combination of multiple diagnostic methods significantly increased the specificity (Supplementary Table S4). For the patients with HPV-16/18, HPV screening combined with either TCT or TS is recommended, which increased the specificity from 40.7% to 98.9% (p<0.0001) and 97.9% (p<0.0001), respectively. For women with HPV-others, we recommend HPV screening plus TCT, which showed better performance than HPV screening plus TS (96.0% vs. 91.6%, p<0.0001). Furthermore, the patients with HPV-16/18 and the patients with HPV-others who showing TCT-positive result should be referred to cervical biopsy under colposcopy or colposcopy only as the follow-up diagnostic test. The combination of three methods have shown higher specificity compared with HPV screening combined with colposcopy (Supplementary Table S6). HPV screening in combination of TCT is recommended as guidelines for cervical cancer screening by some countries 12-14,29. Our results are consistent with that and indicate the importance and necessary of the secondary diagnostic test.

In the regions with sufficient medical resources, the combination of multiple diagnostic methods can easily increase the sensitivity and specificity in the detection of cervical lesions (Figure 4). In the rural areas of China with low medical resources, however, the combination of multiple diagnostic methods mainly limited by economic reasons and the lack of pathology laboratory. In the workflow for cervical lesion detection in these regions, TS is recommended to replace TCT as the secondary method. HPV screening combined with TS has showed comparable specificity compared with in combination with TCT (Supplementary Table S3, S4, and S5). TS is a novel electro-optical device that can provide a real-time result without the need for cytological or molecular analysis. It is a portable device which can be operated by medical personnel following the training manual. The cost of TS is affordable compared with other detection methods (Supplementary Table S7). Among the recommended diagnostic methods, the combination of HPV and TS represents the most cost-effective strategy for the detection of cervical lesion, as detailed in Supplementary Table S8 and illustrated in the Supplementary Figure. Currently, TS has been considered as important diagnostic method for cervical cancer screening in China 30-33.

Our study has several strengths and limitations. One of the strengths is the large and representative sample of women who underwent cervical biopsy at The First People's Hospital of Foshan, which is a tertiary referral center for cervical cancer in the region. This increases the generalizability and applicability of our findings to other similar settings. Another strength is that we compared the performance and accuracy of different methods and combinations using various indicators, such as specificity, sensitivity, and AUC. This provides a comprehensive and objective evaluation of the methods and combinations for cervical cancer screening and detection. One of the limitations of our study is the retrospective design, which may introduce selection bias and confounding factors. For example, some women may have been referred to our hospital because of abnormal results from other diagnostic methods, which may affect the performance and accuracy of the methods and combinations. Another limitation is that we did not include other factors that may influence the outcome of cervical cancer screening and detection, such as age, sexual behavior, smoking, contraceptive use, and immunization status. These factors may modify the association between the methods and combinations and the cervical lesions.

Based on our results, we suggest some possible directions for future research and clinical practice. Future research should use a prospective design or a randomized controlled trial to compare the effectiveness and cost-effectiveness of different methods and combinations for cervical cancer screening and detection. Future research should also include other factors that may affect the outcome of cervical cancer screening and detection, such as demographic, behavioral, and immunological factors. Future research should also explore the feasibility and acceptability of different methods and combinations for cervical cancer screening and detection in different regions with different levels of development. Clinical practice should adopt the combination of multiple diagnostic methods as the standard strategy for cervical cancer screening and detection, depending on the medical resources of the local health care center. Clinical practice should also provide adequate counseling and education for women who undergo cervical cancer screening and detection, and ensure timely referral and treatment for women who have positive results.

## Conclusion

In conclusion, our study compared the efficiency of different methods and combinations including TS, HPV screening, TCT and colposcopy for detecting cervical lesions among 2286 women who underwent cervical biopsy at The First People's Hospital of Foshan. Our study provides recommendations for the optimal screening and detection strategies for cervical lesions in different regions with different levels of development. Our study contributes to the advancement of knowledge and the improvement of public health by offering a feasible and effective solution for cervical cancer prevention and control, especially in low-resource settings. As a non-invasive, easily operated, and portable device, TS is a promising tool to replace TCT as the triage diagnostic method for detecting cervical lesions in the health care center with insufficient medical resources.

## Supplementary Material

Supplementary figure.

Supplementary tables.

## Figures and Tables

**Table 1:** The 2x2 contingency tables used in this study

		Diagnosis		
	Outcome	CIN2+	<CIN2	Sum
Prediction	CIN2+	TP	FP	TP+FP
	<CIN2	FN	TN	FN+TN
	Sum	TP+FN	FP+TN	

**Table 2:** Characteristics distribution of the study population (n = 2286)

		Cervical Biopsy (n=2286)				
Variables	n (%)	Normal	CIN1	CIN2	CIN3	Cancer
**Age (n=2286)**						
<=30	361 (15.8%)	154 (42.7%)	135 (37.4%)	37 (10.2%)	32 (8.9%)	3 (0.8%)
>30&<=50	1508 (66.0%)	642 (42.6%)	555 (36.8%)	121 (8.0%)	165 (10.9%)	25 (1.7%)
>50&<=65	377 (16.5%)	173 (45.9%)	113 (30.0%)	29 (7.7%)	32 (8.5%)	30 (8.0%)
>65	40 (1.7%)	14 (35.0%)	5 (12.5%)	0 (0.0%)	9 (22.5%)	12 (30.0%)
**TruScreen (n=489)**						
TS-negative	343 (70.1%)	306 (89.2%)	32 (9.3%)	2 (0.6%)	3 (0.9%)	0 (0.0%)
TS-positive	146 (29.9%)	102 (69.9%)	28 (19.2)	9 (6.2%)	6 (4.1%)	1 (0.7%)
**Cytology (n=2200)**						
NILM	947 (43.0%)	681 (71.9%)	202 (21.3%	35 (3.7%)	25 (2.6%)	4 (0.4%)
ASC-US	479 (21.8%)	146 (30.5%)	243 (50.7%)	45 (9.4%)	35 (7.3%)	10 (2.1%)
LSIL	463 (21.0%)	102 (22.0%)	280 (60.5%)	53 (11.4%)	27 (5.8%)	1 (0.2%)
ASC-H	69 (3.1%)	13 (18.8%)	29 (42.0%)	5 (7.2%)	20 (29.0%)	2 (2.9%)
HSIL	236 (10.7%)	16 (6.8%)	32 (13.6%)	43 (18.2%)	124 (52.5%)	21 (8.9%)
Cancer	6 (0.3%)	0 (0.0%)	0 (0.0%)	0 (0.0%)	3 (50.0%)	3 (50.0%)
**HPV Infection (n=2202)**						
HPV-negative	633 (28.7%)	491 (77.6%)	114 (18.0%)	12 (1.9%)	8 (1.3%)	8 (1.3%)
HPV-positive (Other)	1117 (50.7%)	361 (32.3%)	522 (46.7%)	117 (10.5%)	103 (9.2%)	14 (1.3%)
HPV-positive (16/18)	452 (20.5%)	108 (23.9%)	149 (33.0%)	54 (11.9%)	116 (25.7%)	25 (5.5%)
**Colposcopy (n=2279)**						
Normal	881 (38.7%)	709 (80.5%)	142 (16.1%)	20 (2.3%)	8 (0.9%)	2 (0.2%)
LSIL	897 (39.4%)	225 (25.1%)	529 (59.0%)	86 (9.6%)	51 (5.7%)	6 (0.7%)
HSIL	438 (19.2%)	49 (11.2%)	129 (29.5%)	81 (18.5%)	168 (38.4%)	11 (2.5%)
Cancer	63 (2.8%)	0 (0.0%)	1 (1.6%)	0 (0.0%)	11 (17.5%)	51 (81.0%)

CIN: cervical intraepithelial neoplasia; NILM: intraepithelial lesion or malignancy; ASC-US: atypical squamous cells of undermined significance; LSIL: low-grade intraepithelial lesion; ASC-H: high-grade squamous intraepithelial lesion; HSIL: high-grade intraepithelial lesion.

**Table 3:** Performance comparison of different methods and combinations by ROC analysis for detecting CIN2+ cervical lesions with different HPV status

	HR-HPV	HPV-16/18	HPV-others
Method	AUC (95% CI)	AUC (95% CI)	AUC (95% CI)
TS	0.742 (0.647 - 0.838)	0.663 (0.501 - 0.825)	0.821 (0.743 - 0.900)
TCT	0.717 (0.693 - 0.740)	0.720 (0.685 - 0.754)	0.724 (0.693 - 0.755)
HPV	0.643 (0.627 - 0.658)	0.788 (0.762 - 0.815)	0.650 (0.627 - 0.672)
COL	0.775 (0.753 - 0.797)	0.793 (0.761 - 0.825)	0.751 (0.720 - 0.781)
TS+TCT	0.789 (0.693 - 0.885)	0.727 (0.567 - 0.886)	0.850 (0.761 - 0.938)
TS+HPV	0.866 (0.810 - 0.923)	0.888 (0.831 - 0.945)	0.901 (0.841 - 0.962)
TS+COL	0.847 (0.771 - 0.923)	0.851 (0.735 - 0.966)	0.860 (0.769 - 0.951)
TCT+HPV	0.785 (0.765 - 0.805)	0.870 (0.847 - 0.893)	0.789 (0.764 - 0.815)
TCT+COL	0.807 (0.784 - 0.830)	0.827 (0.794 - 0.860)	0.798 (0.768 - 0.829)
HPV+COL	0.811 (0.791 - 0.831)	0.881 (0.858 - 0.905)	0.797 (0.770 - 0.824)
TS+TCT+HPV	0.884 (0.828 - 0.941)	0.906 (0.848 - 0.964)	0.908 (0.845 - 0.971)
TS+TCT+COL	0.865 (0.786 - 0.945)	0.865 (0.746 - 0.984)	0.879 (0.785 - 0.974)
TS+HPV+COL	0.921 (0.877 - 0.966)	0.955 (0.909 - 1.000)	0.926 (0.872 - 0.980)
TCT+HPV+COL	0.845 (0.826 - 0.864)	0.904 (0.882 - 0.927)	0.839 (0.813 - 0.864)
TS+TCT+HPV+COL	0.927 (0.881 - 0.972)	0.956 (0.910 - 1.000)	0.930 (0.875 - 0.986)

CIN: cervical intraepithelial neoplasia; AUC: area under the curve; CI: confidence interval; TS: TruScreen; TCT: Thinprep cytological test; HPV: human papillomavirus; COL: colposcopy.

**Table 4:** Performance comparison of different methods and combinations for detecting CIN2+ cervical lesions with different HPV status

	HR-HPV		HPV-16/18		HPV-others	
Method	Sensitivity	Specificity	Sensitivity	Specificity	Sensitivity	Specificity
TS	76.2%	72.2%	60.0%	72.6%	92.3%	72.0%
TCT	48.5%	94.8%	47.4%	96.6%	52.0%	94.7%
HPV	93.9%	34.7%	87.4%	70.2%	89.3%	40.7%
COL	65.1%	90.0%	65.9%	92.7%	59.5%	90.6%
TS+TCT (or†)	81.0%	72.0%	70.0%	72.6%	92.3%	71.8%
TS+TCT (and‡)	19.0%	99.4%	10.0%	100.0%	23.1%	99.3%
TS+HPV (or)	100.0%	47.9%	100.0%	66.1%	100.0%	51.0%
TS+HPV (and)	66.7%	90.6%	40.0%	97.9%	76.9%	91.6%
TS+COL (or)	90.5%	71.6%	90.0%	72.0%	92.3%	71.5%
TS+COL (and)	28.6%	99.1%	30.0%	99.7%	30.8%	99.3%
TCT+HPV (or)	97.3%	33.6%	94.3%	67.8%	95.4%	39.4%
TCT+HPV (and)	45.1%	96.1%	40.2%	98.9%	44.4%	96.0%
TCT+COL (or)	70.6%	87.6%	71.8%	90.9%	68.7%	87.9%
TCT+COL (and)	41.4%	97.2%	42.1%	98.5%	40.5%	97.3%
HPV+COL (or)	96.7%	33.1%	93.3%	66.9%	94.3%	38.9%
HPV+COL (and)	60.6%	91.6%	60.1%	95.9%	54.6%	92.5%
TS+TCT+HPV (or)	100.0%	47.9%	100.0%	66.1%	100.0%	51.0%
TS+TCT+HPV (and)	19.0%	99.4%	10.0%	100.0%	23.1%	99.3%
TS+TCT+COL (or)	90.5%	71.4%	90.0%	72.0%	92.3%	71.3%
TS+TCT+COL (and)	9.5%	99.6%	10.0%	100.0%	7.7%	99.5%
TS+HPV+COL (or)	100.0%	47.6%	100.0%	65.8%	100.0%	50.8%
TS+HPV+COL (and)	23.8%	99.1%	20.0%	99.7%	23.1%	99.3%
TCT+HPV+COL (or)	97.7%	32.4%	95.2%	65.3%	96.1%	38.0%
TCT+HPV+COL (and)	39.2%	97.5%	37.3%	99.1%	36.7%	97.7%
TS+TCT+HPV+COL (or)	100.0%	47.6%	100.0%	65.8%	100.0%	50.8%
TS+TCT+HPV+COL (and)	9.5%	99.6%	10.0%	100.0%	7.7%	99.5%

CIN: cervical intraepithelial neoplasia; CI: confidence interval; TS: TruScreen; TCT: Thinprep cytological test; HPV: human papillomavirus; COL: colposcopy. †: The “or” rule. The diagnosis gives a positive prediction if at least one method shows a positive result. A negative diagnosis is considered only if all methods giving the negative predictions. ‡: The “and” rule. The diagnosis gives a positive prediction only if all methods show the positive predictions. A negative diagnosis is considered if at least one method gives a negative result.

**Table 5:** The recommended diagnostic methods in two workflows for cervical lesion detection

	Method	Advantages	Disadvantages
Optimal workflow	HPV	High sensitivity; Guideline recommended	-
	HPV+TCT	High specificity; Guideline recommended	Pathology laboratory is needed
	HPV+TS	Comparable specificity; Easy operation	TS device may not be introduced in certain regions
	HPV+TCT or TS+COL or Biopsy under COL	Golden standard	Colposcopy specialist is needed
Recommended workflow without pathology laboratory	HPV	High sensitivity; Guideline recommended	-
	HPV+TS	Comparable specificity; Easy operation	TS device may not be introduced in certain regions
	HPV+TS+COL	Golden standard	Colposcopy specialist is needed

TS: TruScreen; TCT: Thinprep cytological test; HPV: human papillomavirus; COL: colposcopy.

## Abbreviations

HPV: human papillomavirus
CIN: cervical intraepithelial neoplasia
TS: TruScreen
TCT: thinprep cytologic test
WHO: World Health Organization
HR-HPV: high-risk human papillomavirus
VIA/VILI: visual inspection of acetic acid or lugol's iodine
CIN2+: cervical lesion CIN2 or worse
TZ: transformation zone
TBS: the Bethesda system
NILM: negative for intraepithelial lesion or malignancy
ASC-US: atypical squamous cells of undermined significance
LSIL: low-grade squamous intraepithelial lesion
ASC-H: atypical squamous cells-cannot exclude high-grade squamous intraepithelial lesion
HSIL: high-grade squamous intraepithelial lesion
PCR: polymerase chain reaction
TPR: true positive rate
TNR: true negative rate
TP: true positive
TN: true negative
FN: false negative
FP: false positive
ROC: receiver operating characteristic
AUC: area under the curve
