# Supplementary material for: Optimal Screening and Detection Strategies for Cervical Lesions: A Retrospective Study
Source: J Cancer. 2024 May 13;15(11):3612–24. doi: 10.7150/jca.96128 (PMC11134435; doi:10.7150/jca.96128)
Supplement: Supplementary file 1 — Supplementary figure. [file jcav15p3612s1.pdf]

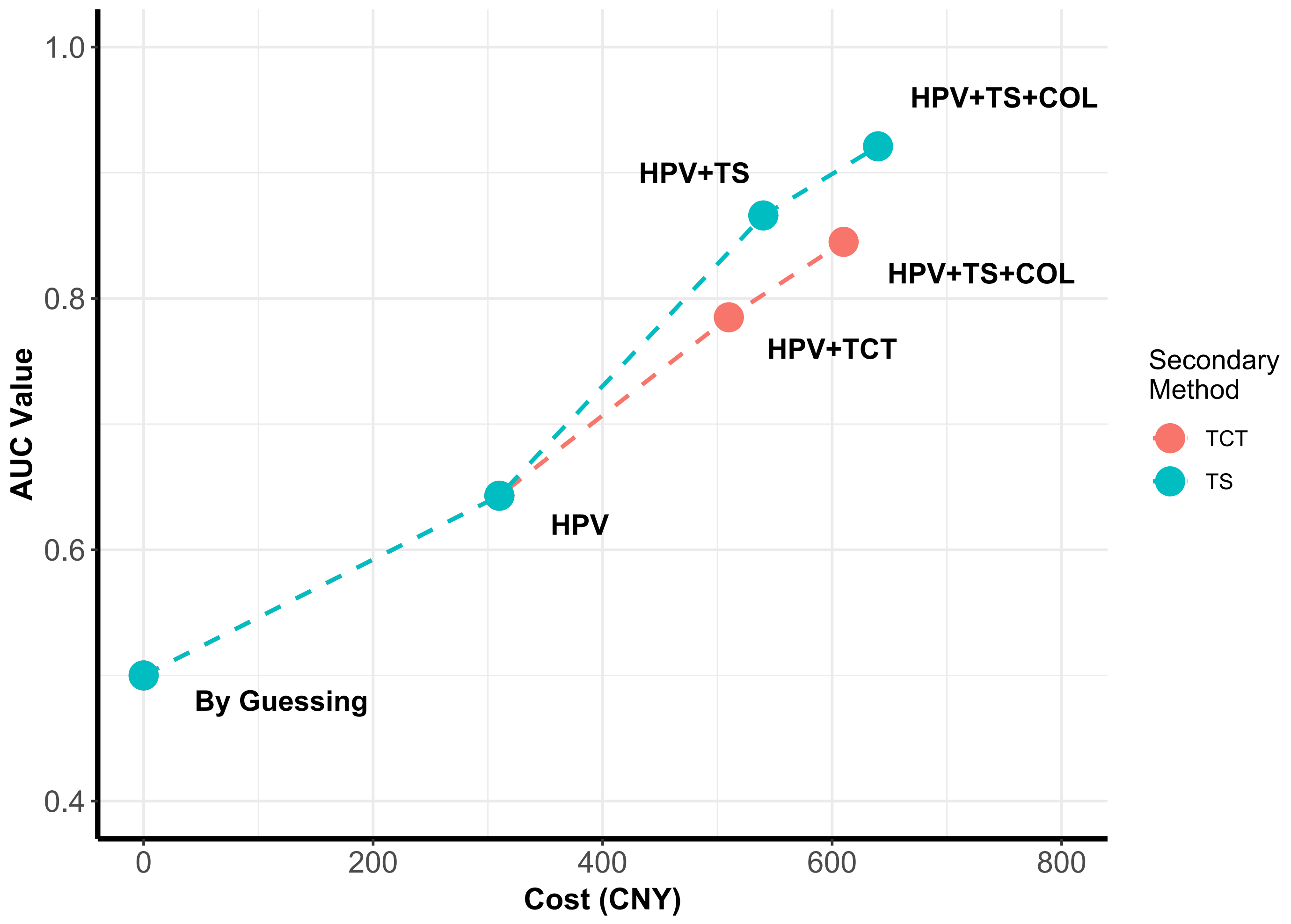

Supplementary Figure. Association between cost and AUC values of the recommended methods for detecting cervical lesions.
